# Supplementary material for: Prospects and challenges for squeezing-enhanced optical atomic clocks
Source: Nat Commun. 2020 Nov 24;11:5955. doi: 10.1038/s41467-020-19403-7 (PMC7686368; doi:10.1038/s41467-020-19403-7)
Supplement: Supplementary file 1 — Supplementary Information [file 41467_2020_19403_MOESM1_ESM.pdf]

Supplementary information: Prospects and challenges for squeezing-enhanced optical atomic clocks

Marius Schulte,[1] Christian Lisdat,[2] Piet O. Schmidt,[2,3] Uwe Sterr,[2] and Klemens Hammerer[1]

[1]*Institute for Theoretical Physics and Institute for Gravitational Physics (Albert-Einstein-Institute), Leibniz University Hannover, Appelstrasse 2, 30167 Hannover, Germany*

[2]*Physikalisch-Technische Bundesanstalt (PTB), Bundesallee 100, 38116 Braunschweig, Germany*

[3]*Institute for Quantum Optics, Leibniz University Hannover, Welfengarten 1, 30167 Hannover, Germany*

(Dated: October 1, 2020)

I. OPTIMAL INTERROGATION TIME

In this section we additionally show the optimal Ramsey times corresponding to Fig. 3(a) of the main text. These are the interrogation times which minimize the model based overall instability, according to Eq. (2) of the main text, for each N. The results are shown in Fig. 1. Overall they follow the same general trend as the instabilities presented in the main text. This is again due to the fact that in the regime of large particle number and long dead times the instability is limited by the tradeoff between Dick effect and CTL which reaches it's minimum at an N-independent interrogation time. For smaller particle numbers the optimal interrogation time actually depends on N. There, spin squeezed states require reduced Ramsey times compared to the uncorrelated states as the minimum between QPN and CTL shifts to smaller values of T_R when the projection noise is reduced. Considering a fixed particle number one finds that as the dead time increases, a longer optimal Ramsey time is required. This is because the observed fraction of the interrogation cycle is increased in order to reduce the Dick effect. In the same way, a shorter dead time is accompanied by a reduced $T_{\mathrm{R,opt}}$. A reduction of the dead time therefore also reduces the relevance of fringe-hops. Looking at figure 4 of the main text, the lowered $T_{\mathrm{R,opt}}$ means moving further left into the blue region of fringe-hop free clock operation. However fringe-hops remain relevant for very small ensembles, $N < 10$, even at $T_\mathrm{D} = 0$ because of the extremely strong influence of the quantum projection noise, see [1].

Again, the optimized squeezing strength here corresponds to weakly squeezed states with the CTL basically unchanged as compared to coherent spin states. We further note that for the laser stability assumed here all optimal interrogation times are on the order of a few seconds.

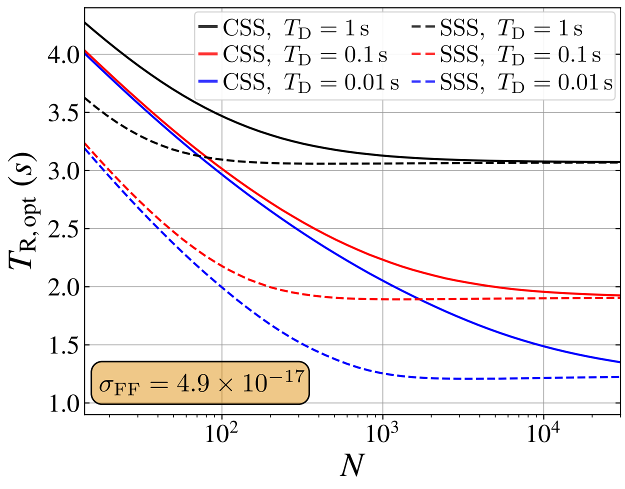

FIG. 1. Optimal Ramsey interrogation times to the results of Figure 3(a) in the main text. Based on the logic of the main text the optimal interrogation times follow the same overall trend as the instability. For the chosen laser noise parameters (see main text for details) they are all on the order of a few seconds.

II. NUMERICAL SIMULATIONS

Building on [1] our numerical analysis simulates the closed feedback dynamics of an atomic clock. First, a time series of the laser frequency noise is generated, corresponding to a given noise characteristic. The noise in each cycle, consisting of the mean differential frequency noise $\delta\nu$ in an interval of the measurement duration T_R and a further interval of length T_D, is generated by discrete stochastic processes. White frequency noise corresponds to independent Gaussian random numbers in each time interval, flicker frequency noise can be generated by a sum of damped random walks, which result in an approximate $1/f$ spectrum over all relevant time scales of the simulation, and random walk of frequency noise results from an ordinary random walk. By specifying a noise spectrum with these three correlation types, whose strengths can be characterized by their respective contributions to an Allan variance, a concrete realization of this process is generated as a sum of the individual noise traces. Based on this, the feedback loop can be simulated. For this purpose, a stochastic measurement result of the atomic reference is generated within each interrogation interval according to the differential phase noise $\delta\phi = 2\pi\delta\nu T_\mathrm{R}$. For uncorrelated atoms the measurement outcome corresponds to a Binomially distributed random number based on to the individual ex-

citation probability $p = \frac{1+\sin(\delta\phi)}{2}$, which is identical for each atom. For spin squeezed states the measurement result is $\mathcal{M} = \langle S_x \rangle \left((1 + \frac{\Delta S_x}{\langle S_x \rangle}\mathcal{N}) \sin(\delta\phi) + \frac{\Delta S_y}{\langle S_x \rangle}\mathcal{N} \cos(\delta\phi) \right)$ where $\mathcal{N}$ are standard-normally distributed random variables with expectation value 0 and variance 1. We make a Gaussian approximation to the measurement statistics of spin squeezed states at the end of the Ramsey sequence at $N \geq 20$. This means that we neglect any cumulants of order three or higher in the probability distributions for measurement results of $S_{x,y,z}$. Spin variances are as given in the methods section. The measurement results are limited by the fact that their value range may not exceed $-N/2$ to $N/2$ and each result is statistically independent from all others. Using these outcomes the feedback servo generates estimates of the frequency deviations via linear estimation, which uses the linear dependence between the measurement result and the phase, valid for small relative phase fluctuations, to determine the corresponding frequency errors given otherwise only the slope of the signal at $\phi = 0$. Then the feedback corrections are applied using a (double) integrator [1, 2] to stabilize the output frequency. From the stabilized frequency trace the stability at $\tau = 1\,\mathrm{s}$ is extracted by numerically fitting the pre-factor to the asymptotic $1/\sqrt{\tau}$ scaling, reached typically after a few thousand cycles of clock operations in simulations with a total of 8×10^5 cycles. For all simulation results presented in the main text we used moderate feedback with $g = 0.4$ and the squeezing strength was optimized beforehand for each N to give the lowest instability without dead time.

[1] I. D. Leroux, N. Scharnhorst, S. Hannig, J. Kramer, L. Pelzer, M. Stepanova, and P. O. Schmidt, "On-line estimation of local oscillator noise and optimisation of servo parameters in atomic clocks," *Metrologia*, vol. 54, pp. 307–321, Apr 2017.

[2] E. Peik, T. Schneider, and C. Tamm, "Laser frequency stabilization to a single ion," *Journal of Physics B: Atomic, Molecular and Optical Physics*, vol. 39, pp. 145–158, Dec 2005.
